# Abdominal Cocoon Syndrome With Greater Omental Hypoplasia: A Rare Anatomical Insight and Diagnostic Challenge

**DOI:** 10.1002/ccr3.72147

**Published:** 2026-02-26

**Authors:** Sohrab Sayyadi, Keihan Shabankhani, Mohammad Mahdi Sargazi Moghaddam, Seyed Mohammad Sakhaei, Mina Alvandipour, Seyed Muhammad Mehdi Ghaffari Hamedani, Hossein Farsavian, Mohammad Javad Najafi

**Affiliations:** ^1^ Department of Surgery, Faculty of Medicine Mazandaran University of Medical Sciences Sari Iran; ^2^ Department of Anesthesiology, Faculty of Medicine Mazandaran University of Medical Sciences Sari Iran; ^3^ Student Research Committee Mazandaran University of Medical Sciences Sari Iran; ^4^ Department of Radiology and Nuclear Medicine, Faculty of Medicine Mazandaran University of Medical Sciences Sari Iran; ^5^ Clinical Research Development Unit of Imam Khomeini Hospital Mazandaran University of Medical Science Sari Iran

**Keywords:** abdominal cocoon syndrome, adhesiolysis, case report, omental hypoplasia, sclerosing encapsulating peritonitis, small bowel obstruction

## Abstract

Abdominal cocoon syndrome, or idiopathic sclerosing encapsulating peritonitis (SEP), is an uncommon cause of small bowel obstruction marked by the encasement of bowel loops within a dense fibrocollagenous membrane. Due to its rarity and nonspecific presentation, diagnosis is frequently delayed, particularly in patients without prior abdominal surgery or known risk factors. We report a diagnostically challenging case of a 53‐year‐old male with an 18‐month history of recurrent gastrointestinal obstruction, including bilious vomiting, oral intolerance, and epigastric pain. Multiple prior evaluations yielded inconclusive findings. Contrast‐enhanced computed tomography raised suspicion for SEP, prompting exploratory laparotomy. Intraoperative findings confirmed SEP, along with a rare anatomical anomaly—hypoplasia of the greater omentum. Surgical excision of the encasing membrane and adhesiolysis resulted in complete symptom resolution, with no recurrence at six‐month follow‐up. This case underscores the importance of clinical vigilance in recognizing abdominal cocoon syndrome and highlights the first reported co‐occurrence of SEP with greater omental hypoplasia. Surgical exploration not only confirmed the diagnosis but also achieved full recovery. The embryologic implications of omental hypoplasia may offer new insights into SEP pathophysiology and merit further investigation.

## Key Clinical Message

Abdominal cocoon syndrome is a rare cause of bowel obstruction. Its co‐occurrence with greater omental hypoplasia highlights a unique embryologic anomaly and reinforces the diagnostic and therapeutic value of early surgical exploration in unexplained, recurrent gastrointestinal symptoms.

## 1 Introduction

Abdominal cocoon syndrome, or idiopathic sclerosing encapsulating peritonitis (SEP), is a rare, often under‐recognized cause of small bowel obstruction. Described in the early 1900s, it involves the small intestine encased by a dense fibrocollagenous membrane. Its exact cause is unknown, but it mainly affects young males in tropical and subtropical areas [1, 2].

Abdominal cocoon syndrome, due to its rarity and nonspecific symptoms, is often misdiagnosed or missed and is usually found only during surgery. Symptoms range from chronic abdominal pain to acute bowel obstruction [3].

Without prior abdominal surgery or risk factors, suspicion is usually low, and the condition may be mistaken for common causes like adhesive disease, internal hernia, or vascular compression [4].

Preoperative imaging frequently results in inconclusive findings when diagnosing this condition; even contrast‐enhanced computed tomography may not provide definitive results, but it remains valuable for excluding other differential diagnoses. Due to diagnostic challenges and the potential curative role of surgery, heightened clinical vigilance is essential, especially in patients with recurrent, unexplained gastrointestinal obstructions [5].

Surgical intervention, especially laparotomy, remains the cornerstone and most effective treatment modality for SEP [6].

Nevertheless, its utilization is a subject of ongoing discourse concerning suitable indications, optimal timing, and procedural techniques. These ambiguities arise from less‐than‐ideal surgical outcomes observed in certain instances, where patients may experience postoperative complications such as recurrent small bowel obstruction or the development of new adhesions [6].

We present a middle‐aged male with a prolonged history of nonspecific gastrointestinal symptoms, diagnosed with SEP only after exploratory laparotomy. This report highlights a challenging case where surgery was crucial for diagnosis.

## 2 Case History/Examination

A 53‐year‐old man was referred with recurrent episodes over 18 months, involving oral intolerance, nausea, bilious vomiting, and intestinal obstruction. Each episode included severe epigastric pain worsened by eating and up to eight episodes of bilious emesis. He reported complete oral intake intolerance during these times.

He denied fever, chills, or rigors. The episodes followed a consistent pattern, prompting multiple consultations that provided temporary relief without diagnosis. Prior records showed normal labs, no air‐fluid levels, and unremarkable endoscopies.

His medical history was otherwise unremarkable, with no chronic illnesses, prior surgeries, significant family history, or alcohol use, and no unintentional significant weight loss over the last 18 months. The patient reported initiating opium use in response to recurrent episodes of abdominal pain, which subsequently progressed to opioid dependence. At the time of referral, he was enrolled in a methadone maintenance program and was not taking any other regular medications.

During a prior episode, the medical team suspected superior mesenteric artery (SMA) syndrome and referred the patient to our facility for continued evaluation and management.

On presentation, he appeared ill but was alert and cooperative. Vital signs were stable: temperature 37°C, blood pressure 125/75 mmHg, heart rate 95 bpm, oxygen saturation 99%. Ocular exam showed no pallor or scleral icterus. Cardio‐pulmonary eval was normal, with regular heart sounds and clear lungs. Abdominal exam revealed mild generalized distension. The soft abdomen was diffusely tender, especially in the epigastric area, without rebound, guarding, or organomegaly. Bowel sounds were hypoactive. Laboratory results showed normal electrolytes and blood glucose. Renal markers were elevated (BUN: 65 mg/dL, creatinine: 1.7 mg/dL), indicating dehydration‐related pre‐renal azotemia. Mild leukocytosis (WBC: 10.9 × 10^3^/μL) and thrombocytosis (436 × 10^3^/μL) were observed, with normal hemoglobin and coagulation.

**FIGURE 1:**
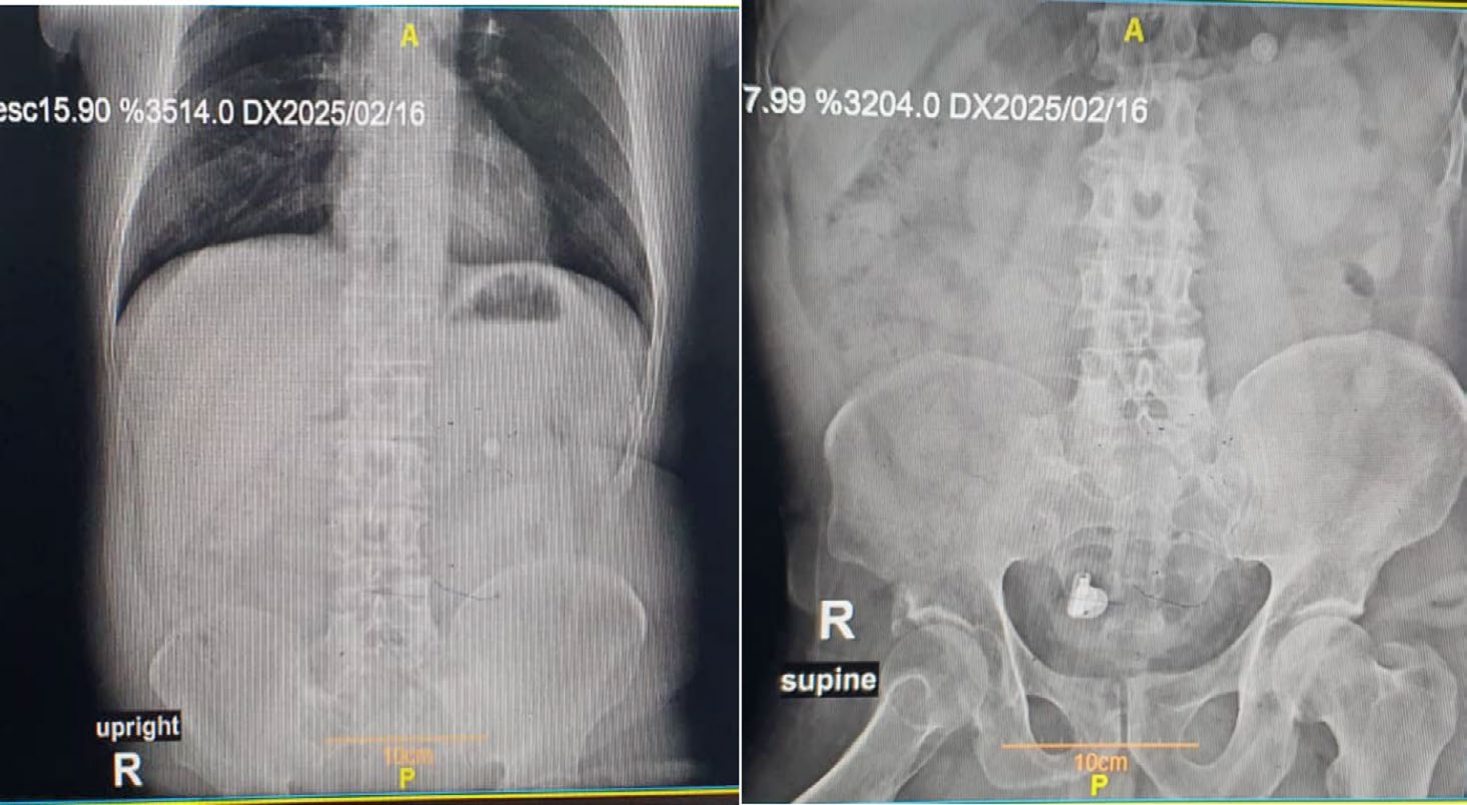
Abdominal X‐ray.

**FIGURE 2:**
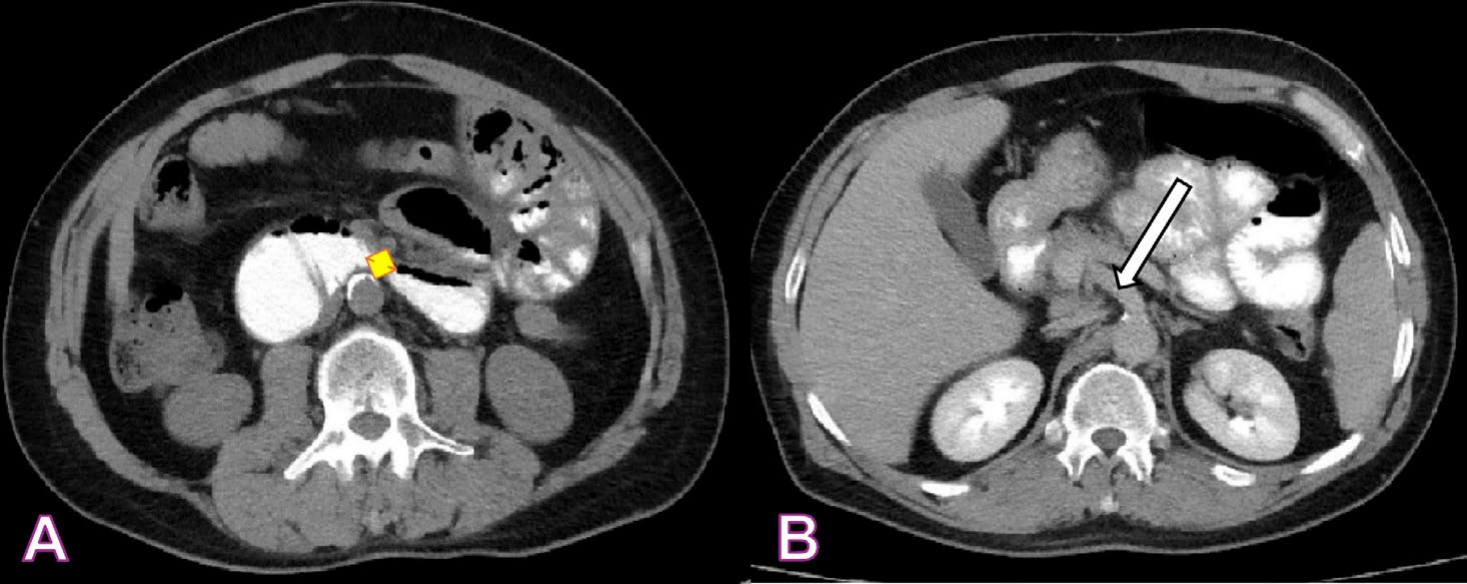
Contrast‐enhanced computed tomography (axial plane). (A) Aortomesenteric distance exceeding 12 mm; (B) Superior Mesenteric Artery indicated by a white arrow.

**FIGURE 3:**
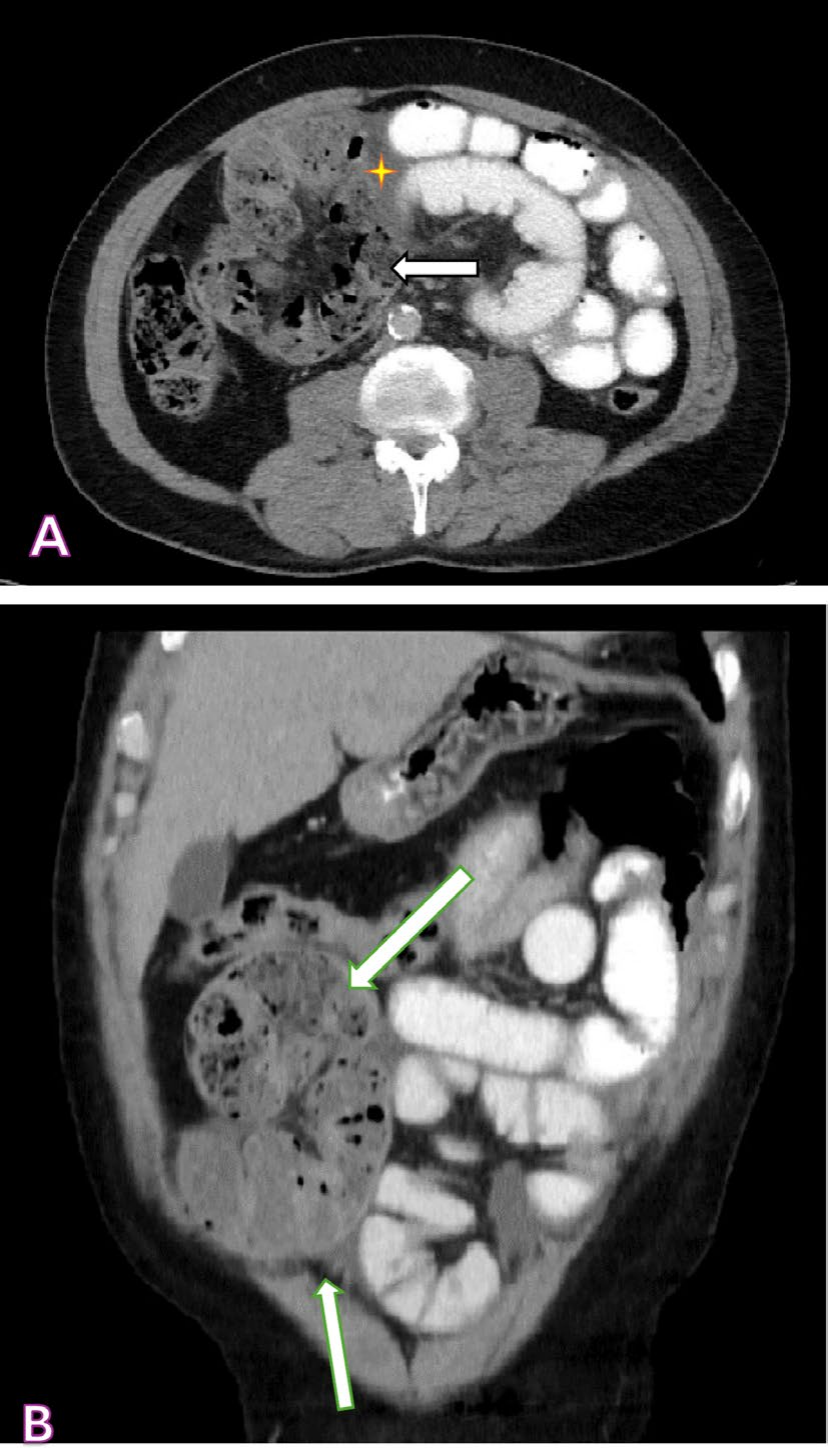
Encapsulating peritoneal sclerosis (EPS), characterized by fibrosis of the peritoneum (white arrow) and the encasement of the small intestine, with minimal ascites (yellow star). (A) Axial plane; (B) Coronal plane.

Abdominal radiography revealed no diagnostic abnormalities (Figure 1). Consequently, a contrast‐enhanced computed tomography (CT) scan was performed. CT findings excluded SMA syndrome (Figure 2) and raised suspicion for SEP based on the presence of peritoneal fibrosis and mild ascites (Figure 3).

## 3 Differential Diagnosis, Investigations, and Treatment

Despite support, the patient showed symptoms of proximal intestinal obstruction, such as increasing abdominal distension and oral intolerance. Due to suspicion of abdominal cocoon syndrome and radiologic findings, the surgical team chose to perform an exploratory laparotomy. Diagnostic laparoscopy was unsafe because of severe distension.

**FIGURE 4:**
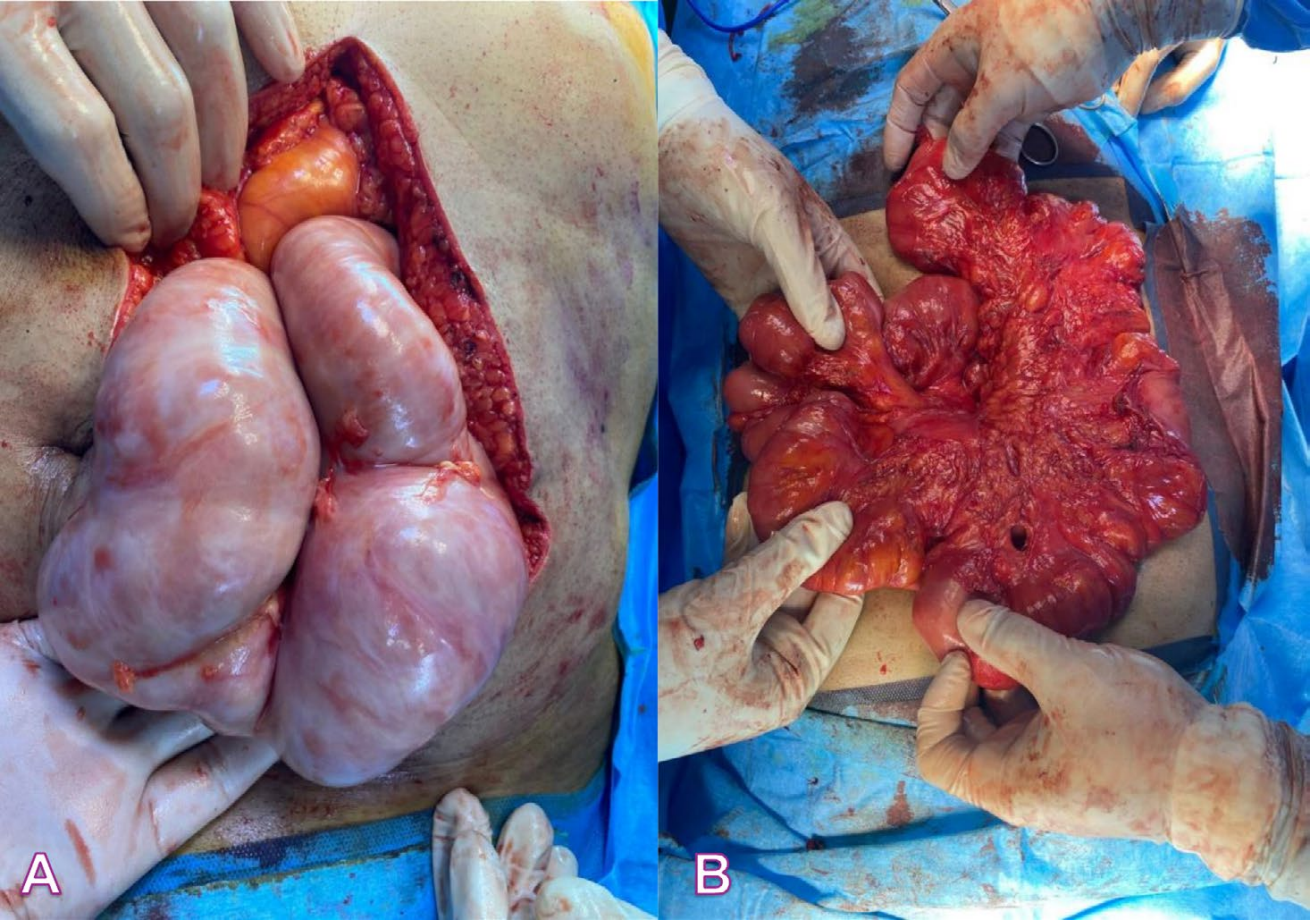
(A) Encapsulated small bowel; (B) Released encapsulated small bowel.

Intraoperatively, hypoplasia of the greater omentum was observed, with the entire small intestine encased in a thick fibrous membrane and dense adhesions limiting bowel mobility, consistent with abdominal cocoon syndrome. The membrane was carefully dissected and removed, and adhesiolysis was performed to free the bowel. No resection, ischemia, or perforation was present (Figure 4).

## 4 Conclusion and Results

Postoperatively, the patient recovered uneventfully. Oral intake resumed 24 h later, was well tolerated, and without complications. Abdominal pain resolved, and bowel function normalized. He was discharged in stable condition. At six months, he remained asymptomatic, with no GI complaints, and resumed regular diet and activity.

## 5 Discussion

Abdominal cocoon syndrome is a rare but important diagnosis in patients with chronic or recurrent small bowel obstruction, especially without prior abdominal surgery or underlying issues [7] Initially described in 1907 and clarified in 1978, it is characterized by a dense fibrocollagenous membrane encasing the small bowel and sometimes other organs, causing mechanical obstruction [8].

The pathogenesis of idiopathic abdominal cocoon syndrome remains poorly understood. Proposed mechanisms include retrograde peritonitis, omental hypoplasia, and mesenteric vascular anomalies [9]. Secondary forms have been linked to factors such as peritoneal dialysis, beta‐blocker therapy, tuberculosis, sarcoidosis, and systemic autoimmune diseases. The idiopathic variant is more commonly observed in tropical and subtropical regions, primarily affecting young males; however, cases have been documented across a broader demographic spectrum [10, 11].

Clinically, the syndrome manifests as recurrent or chronic abdominal pain, nausea, vomiting, and symptoms indicative of subacute intestinal obstruction. Patients often recount intermittent episodes lasting months or years, with spontaneous remission complicating timely diagnosis [12]. In the present case, our patient experienced multiple such episodes over a period of 18 months prior to undergoing definitive surgical management.

Anatomical anomalies like greater omentum hypoplasia are very rare and rarely reported with abdominal cocoon syndrome. The greater omentum, from dorsal mesogastrium, is essential for immune surveillance, angiogenesis, and peritoneal fluid dynamics [13, 14]. Hypoplasia may reflect disrupted mesodermal differentiation or defective peritoneal folding during the 4th to 8th week of gestation [15, 16]. In our case, the absence of a fully developed omental curtain could have impaired mechanical protection and localized immune regulation, potentially contributing to aberrant fibrocollagenous membrane formation. The co‐occurrence of SEP and omental hypoplasia suggests a possible shared embryopathogenic pathway, opening avenues for future exploration into peritoneal morphogenesis and intra‐abdominal immune responses. Recognition of this pairing not only expands the spectrum of SEP presentations but also enhances the surgical understanding of rare peritoneal disorders.

Radiologic diagnosis presents considerable challenges. CT imaging may demonstrate clumped bowel loops encased within a membranous capsule, occasionally referred to as the “cauliflower sign,” in conjunction with peritoneal thickening and ascites [17]. Nevertheless, the sensitivity of imaging modalities remains limited, and a definitive diagnosis is frequently established intraoperatively. In our case, despite the administration of two contrast‐enhanced CT scans, spontaneous encapsulating peritoneal sclerosis (SEP) was not conclusively identified until exploratory laparotomy.

Surgical treatment remains the main approach for symptomatic cases. Complete excision of the fibrous membrane and adhesiolysis usually resolve symptoms with low recurrence rates when extensively removed. Conservative methods may be suitable for carefully selected, minimally symptomatic cases but are often inadequate in idiopathic cases requiring surgery due to progressive obstruction intervention [18].

## 6 Conclusion

This case underscores the diagnostic challenges and clinical significance of abdominal cocoon syndrome in patients presenting with unexplained, recurrent gastrointestinal obstruction. Although rare, the condition should be considered in the differential diagnosis when suggestive clinical features are present and imaging remains inconclusive. Surgical exploration offers both definitive diagnosis and curative treatment, with favorable outcomes when performed in a timely manner. Heightened awareness among clinicians is vital to ensure early recognition and avoid delays in management.

## Author Contributions

**Sohrab Sayyadi:** data curation, investigation, methodology, writing – original draft. **Keihan Shabankhani:** data curation, investigation, writing – original draft. **Mohammad Mahdi Sargazi Moghaddam:** data curation, investigation, writing – review and editing. **Seyed Mohammad Sakhaei:** conceptualization, data curation, investigation, writing – review and editing. **Mina Alvandipour:** investigation, writing – review and editing. **Seyed Muhammad Mehdi Ghaffari Hamedani:** investigation, writing – review and editing. **Hossein Farsavian:** investigation, writing – review and editing. **Mohammad Javad Najafi:** conceptualization, data curation, investigation, project administration, supervision, validation, visualization, writing – original draft, writing – review and editing.

## Funding

The authors have nothing to report.

## Ethics Statement

All procedures conducted were compliant with the ethical standards established by the responsible committee on human experimentation, both institutional and national, and adhered to the Helsinki Declaration of 1975, as amended in 2008. The research protocol received approval from the Research Ethics Committees (Approval Code: IR.MAZUMS.REC.1404.136).

## Consent

Written informed consent was obtained from the patient to document this case report.

## Conflicts of Interest

The authors declare no conflicts of interest.

## Data Availability

Data are available upon reasonable request to the corresponding author, pending ethical committee approval.
